# *Salmonella* aortitis treated with endovascular aortic repair: a case report

**DOI:** 10.1186/1752-1947-6-243

**Published:** 2012-08-15

**Authors:** Carol Strahm, Heidi Lederer, Esther I Schwarz, Esther B Bachli

**Affiliations:** 1 Clinic of Internal Medicine, Uster Hospital, Brunnenstrasse 42, Uster, CH-8610, Switzerland; 2 Division of Internal Medicine, University Hospital Zurich, Zurich, Switzerland; 3 Institute of Surgical Pathology, University Hospital Zurich, Zurich, Switzerland

**Keywords:** *Salmonella* Enteritidis, Aortitis, Mycotic aneurysm, EVAR, Endovascular aortic repair

## Abstract

**Introduction:**

*Salmonella* is a typical cause of aortitis, which is associated with high morbidity and mortality. In infrarenal disease, besides open surgery, endovascular aortic repair as an alternative treatment has been reported. To the best of our knowledge, we report the first successful endovascular aortic repair documented by necropsy to date.

**Case presentation:**

A 67-year-old Caucasian man presented with low back pain, fever and positive blood cultures for *Salmonella* Enteritidis. A computed tomography scan showed an enlargement and intramural hematoma of the infrarenal aortic wall; a *Salmonella* aortitis was suspected and antimicrobial therapy initiated. Because of substantial comorbidities, endovascular aortic repair was favored over open surgery; postoperatively the antibiotic treatment was continued for 12 months. Post-mortem there were neither macroscopic nor microscopic signs of aortitis or graft infection.

**Conclusions:**

We could demonstrate by necropsy that endovascular aortic repair of infrarenal aortitis with prolonged pre- and postinterventional antibiotic therapy for 12 months was a minimally invasive alternative and should be considered in selected clinically stable patients with substantial co-morbidities.

## Introduction

Infectious aortitis is a rare but life-threatening disorder [1]. Usually a combined strategy of antibiotic treatment and surgical debridement is required, although no controlled clinical trials have explored its optimal management. Despite aggressive therapeutic approaches mortality remains high [2-7]. An alternative is combined medical and endovascular management. We report the case of a patient with aortitis due to *Salmonella* Enteritidis that was successfully treated with endovascular aortic repair (EVAR) and antibiotic therapy as confirmed by autopsy.

Infectious aortitis is an inflammatory process within the arterial wall caused by microorganisms. As a complication, microbial arteritis with aneurysm can develop. Possible mechanisms are septic emboli to the vasa vasorum, continuous infection from a focus extending to the arterial wall, direct bacterial inoculation and bacterial seeding in an existing intimal injury or an atherosclerotic plaque [1]. Typical risk factors are male gender, age over 50 years, diabetes, and pathological alterations of the aortic wall (most commonly atherosclerosis) [1]. The most frequently isolated pathogens are *Salmonella* and staphylococcal species [8,9].

Diagnosis is based on the clinical picture, radiographic imaging, and culture results. Because of the nonspecific presentation with back pain or abdominal discomfort and fever, the diagnosis of infectious aortitis requires a high index of suspicion [5]. Nevertheless, an early diagnosis is crucial, because microbial arteritis with aneurysm is associated with a high rate of rupture and subsequent mortality if left untreated. Once the diagnosis is made, a rapid empirical intravenous antibiotic therapy is important while awaiting microbiological data from blood cultures [1,2].

## Case presentation

A 67-year-old Caucasian man presented to the emergency department with back and abdominal pain, fever and malaise. He reported that his wife and granddaughter had previously suffered from mild gastroenteritis. He had a history of colorectal cancer with a solitary metastasis (7 × 7.5cm) of the abdominal wall, and previously underwent aortocoronary bypass surgery and mitral valve reconstruction. On examination, he was febrile (38.5°C axillary) with otherwise normal vital signs. The abdomen was distended with discomfort on deep palpation with a palpable mass in the abdominal wall. The lower spine was tender on percussion.

**Figure 1:**
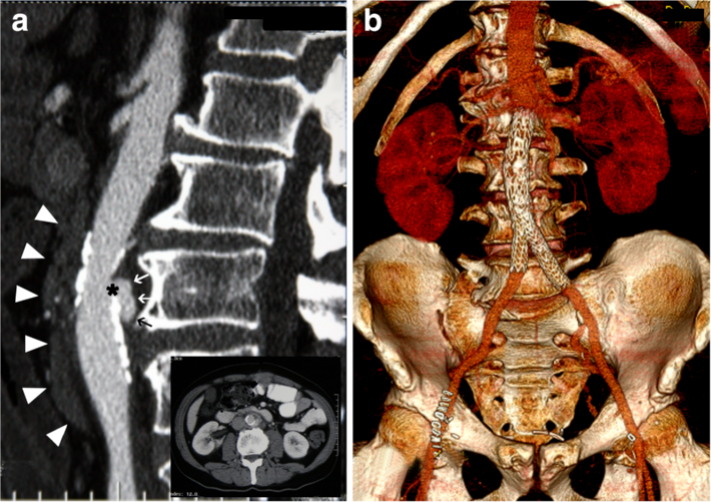
(a) Sagittal reconstruction of the contrast-enhanced initial computed tomography (CT) scan showing typical appearance of a mycotic aneurysm: eccentric saccular pseudo-aneurysm (arrows) of the abdominal aorta (arising from a ‘breach’(*) in the aortic wall) and periaortic soft tissue stranding (arrowheads). (**b**) 3D-reconstruction of the postinterventional computed tomography (CT) scan showing the anatomic location of the aortic stent graft.

Laboratory tests showed an elevated C-reactive protein (99mg/L, normal < 10mg/L) with mild leukocytosis (Table 1). Three out of four pairs of blood cultures grew *Salmonella enterica* serotype Enteritidis (serogroup D). Stool cultures for *Salmonella* were negative. After exclusion of endocarditis, a computed tomographic (CT) scan for an endovascular focus showed an intramural hematoma of the infrarenal aorta (20 x 18 x 6mm) with paraaortic fat stranding as well as atherosclerotic plaques (Figure 1). Thus, the diagnosis of an infectious aortitis caused by *S.* Enteritidis was made and he was treated with ceftriaxone and ciprofloxacin. Open surgery with excision of the inflammatory tissue and extra-anatomical bypass-grafting or in situ reconstruction was not an option because the colon carcinoma had metastasized directly in the potential operation area. Since there was neither evidence of gross aneurysm nor active bleeding, he was initially treated with intravenous antibiotics and after a 16-day course of antimicrobial therapy in the presence of a stable follow-up documented by computed tomography (CT) scan, EVAR with the Gore Excluder (W. L. Gore & Associates GmbH, Putzbrunn, Germany) was performed.

**Table 1:** Laboratory values at hospital admission (19 days before EVAR), ten days before EVAR and shortly before the EVAR with follow-up at discharge and after three, six, 12 and 15 months

	**Day −19**	**Day −10**	**Day 0**	**Day 9**	**Three months**	**Six months**	**12 months**	**15 months**
	**Admission**		**EVAR**	**Discharge**	**Follow-up**			
Hb (g/L)	144	121	116	104	92	100	139	116
Leuk (G/L)	10.8	9.7	6.4	4.9	6.9	6.2	8.1	6.6
Thr (G/L)	181	343	312	276	474	259	241	262
CRP (mg/L)	99	81	22	17	<5	<5	<5	7.7

*CRP* C-reactive protein, *EVAR* endovascular aortic repair, *Hb* hemoglobin; *Leuk* leukocytes; *Thr* thrombocytes.

**Figure 2:**
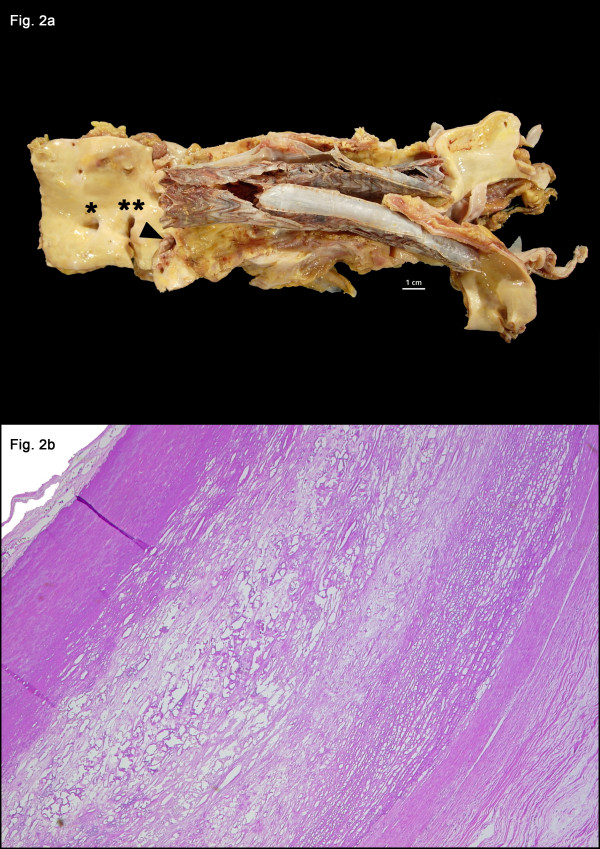
**A series of abdominal computed tomography (CT) scan studies from the patient, the first at the time of diagnosis one week before endovascular aortic repair (EVAR) and regular follow-up computed tomography (CT) scans one week after EVAR and after three, six, 12 and 15 months.** The initial intramural hematoma and soft tissue stranding has completely disappeared three months after EVAR and no signs of persistent or relapsed infection are detectable on follow up scans. Three months after discontinuation of antibiotic therapy (15 month after EVAR) there are no signs of relapse.

The post-interventional clinical course was uneventful. Intravenous antibiotic therapy was continued for another 10 days (altogether 26 days) and then switched to an oral monotherapy with ciprofloxacin for 12 months. Repeated computed tomography (CT) scans (after three, six, 12 and 15 months) and blood analysis (with leukocyte counts and C-reactive protein) were performed during follow-up (Table 1 and Figure 2).

**Figure 3:**
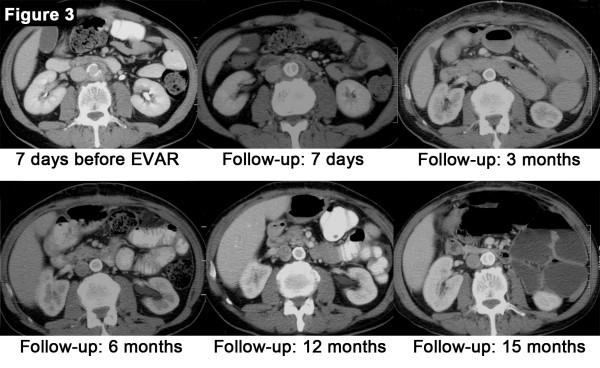
**(a) Aorta abdominalis and common iliac arteries with endoluminal aortic Y-prothesis (Gore Excluder)*in situ*directly distal of the ostia of the renal arteries opened from dorsal.** Orifices from left to right: celiac trunc (*), superior mesenteric artery (**), left renal artery (arrowhead). (**b**) Haematoxylin and Eosin staining of a cross-section of the aortic wall showing enhanced intimal and medial thickening due to fibrosis and atherosclerosis, but no inflammatory infiltrates.

Four months later (16 months after EVAR) the patient died of an ileus. Postmortem the macroscopic examination revealed circumferential atheromatous thickening of the intima and media due to extensive fibrosis and sclerosis (Figure 3a). Microscopically, diffuse fibrosis and sclerosis of the aortic wall in the area of the endoluminal stent graft was described. These findings were unspecific but consistent with lapsed aortitis. There were no signs of residual active or chronic inflammation (Figure 3b).

## Discussion

The mortality of infectious aortitis and mycotic aneurysm is high and depends on rapid diagnostic workup, location of the diseased artery and type of therapy as well as the underlying condition of the patient [4]. In the largest case series with combined surgical and antibiotic treatment, an early mortality of 11% to 36% is documented [6,7]. Medical management without surgery is fatal in greater than 90% of cases [10].

Wang *et al*. [11] reported that 35% of adults above 65 years of age with positive blood cultures for non-typhoid *Salmonella* had aortitis. Common risk factors for invasive non-typhoid salmonellosis are immunodeficiency and solid-organ cancer [12]. Since most patients have severe comorbidity an open surgical procedure carries a high perioperative risk [1].

A less invasive approach, namely, combined medical and endovascular management has been introduced by Semba *et al*. [13]. So far, only case reports and small series of successful EVAR of infectious aortitis have been reported and reviewed; hence, it is still considered an experimental procedure [3,6]. Regardless, EVAR has changed the management of primary mycotic aneurysm over the last decades [14]. At present, it is probably best documented for *Salmonella* infection [15,16].

Based on available data, the early mortality rate associated with stent-grafts appears to be lower than the one associated with conventional surgery. In a recent small multi-institutional case series, EVAR performed in selected patients showed a very good outcome with no in-hospital mortality and only one unrelated late death [15]. However, late aneurysm-related events including mortality and complications seem to be more common than with surgery [6], because there is obviously no possibility to debride the infectious tissue and the infected aortic wall [17]. As the majority of late events resulted from inadequate control of infection, an appropriate preoperative antibiotic treatment preferably with clearance of the bacteremia as well as long-term treatment after EVAR is mandatory [3,6]. Various factors including anatomic location of the infection (infrarenal, paravisceral, or thoracic aorta), the nature (fistula versus mycotic aneurysm), the microbial features, the general condition and the morbidity of the patient should be considered before establishing endovascular treatment as a viable option. It is difficult to define which cases should be treated endoluminally and it seems that the endovascular technique should be used only for specific indications, such as absence of gross purulence, gross infection, aorto-digestive fistula and uncontrolled sepsis and in the presence of a high operative risk [16,18].

Our patient had severe comorbidity and there were neither signs of gross purulence nor fistula. In addition, he responded rapidly to antibiotic therapy. After five days the blood cultures turned negative and several follow-up blood cultures remained negative.

Therefore, we favored an EVAR without delay in this setting.

The optimal antibiotic agent and duration of treatment after EVAR remains controversial and some authors recommend lifelong resumption [15,16]. Prolonged antibiotic therapy is generally considered necessary [6]. In our case, after successful EVAR, an intravenous antibiotic therapy was continued for 10 more days. Since at that time our patient was in a good general condition, we switched to an oral monotherapy with ciprofloxacin, which was resumed for a period of 12 months. The duration of antibiotic therapy was chosen empirically because simultaneously there was maintenance chemotherapy for his colon cancer, which was considered as immunosuppression. Given normal C-reactive protein, leukocyte counts, clinical status and negative computed tomography (CT) the antibiotic therapy was stopped.

In device-associated infections due to gram-negative rods fluoroquinolones should be favored, because quinolones are active against adherent and nongrowing *Escherichia coli *[19]. In addition, in an animal model of implant-associated infection, ciprofloxacin was better than cotrimoxazole against *S.* Dublin [20]. Another reason in favor of quinolones is the intracellular bactericidal activity against non-typhoid *Salmonella in vitro* as shown by Chiu *et al*. [21].

Careful follow-up is of great importance to detect persistent infection, endoleaks and migration of the graft [1,6]. Our patient underwent CT scans three, six, 12 and 15 months after EVAR and was stable at follow-up without signs of therapy failure (Figure 2).

Four months after suspension of antibiotic therapy the patient died in the course of an ileus. As post-mortem examination found no evidence of active inflammation of the aorta or signs of stent failure, one could assume the patient to be cured of the infection.

## Conclusions

To conclude, it was shown that EVAR with prolonged antibiotic treatment with an active antibiotic against adherent bacteria for 12 months can be considered as an efficacious alternative strategy in appropriate patients with infectious aortitis due to *S.* Enteritidis.

## Consent

Written informed consent was obtained from the patient prior to his death for publication of this case report and any accompanying images. A copy of the written consent is available for review by the Editor-in-Chief of this journal.

## Competing interests

The authors declare that they have no competing interests.

## Authors’ contributions

CS analyzed and interpreted the patient data and was the major contributor in writing the manuscript. EIS performed the necropsy and histological examination of the aorta. HL and EBB were contributors in writing the manuscript. All authors read and approved the final manuscript.
